# Engineering Kitaev exchange in stacked iridate layers: impact of inter-layer species on in-plane magnetism

**DOI:** 10.1039/c8sc03018a

**Published:** 2018-12-03

**Authors:** Ravi Yadav, Mohamed S. Eldeeb, Rajyavardhan Ray, Saicharan Aswartham, Mihai I. Sturza, Satoshi Nishimoto, Jeroen van den Brink, Liviu Hozoi

**Affiliations:** a Leibniz Institute for Solid State and Materials Research , IFW Dresden , Helmholtzstr. 20 , 01069 Dresden , Germany . Email: r.yadav@ifw-dresden.de; b Dresden Center for Computational Materials Science (DCMS) , TU Dresden , 01062 Dresden , Germany; c Department of Physics , Technical University Dresden , Helmholtzstr. 10 , 01069 Dresden , Germany

## Abstract

Novel functionalities may be achieved in oxide electronics by appropriate stacking of planar oxide layers of different metallic species, MO_*p*_ and M′O_*q*_. The simplest mechanism allowing the tailoring of the electronic states and physical properties of such heterostructures is of electrostatic nature—charge imbalance between the M and M′ cations. Here we clarify the effect of interlayer electrostatics on the anisotropic Kitaev exchange in H_3_LiIr_2_O_6_, a recently proposed realization of the Kitaev spin liquid. By quantum chemical calculations, we show that the precise position of H^+^ cations between magnetically active [LiIr_2_O_6_]^3–^ honeycomb-like layers has a strong impact on the magnitude of Kitaev interactions. In particular, it is found that stacking with straight interlayer O–H–O links is detrimental to in-plane Kitaev exchange since coordination by a single H-ion of the O ligand implies an axial Coulomb potential at the O site and unfavorable polarization of the O 2p orbitals mediating the Ir–Ir interactions. Our results therefore provide valuable guidelines for the rational design of Kitaev quantum magnets, indicating unprecedented Kitaev interactions of ≈40 meV if the linear interlayer linkage is removed.

## Abstract 2

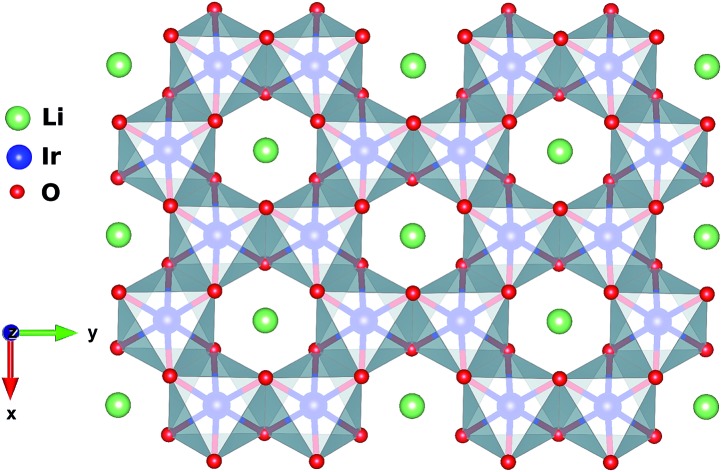
Electrostatic effects involving the inter-layer species are important: the largest Kitaev interactions comewith a more isotropic distribution of inter-layer cations around a given ligand.

## Introduction

**Fig. 1:**
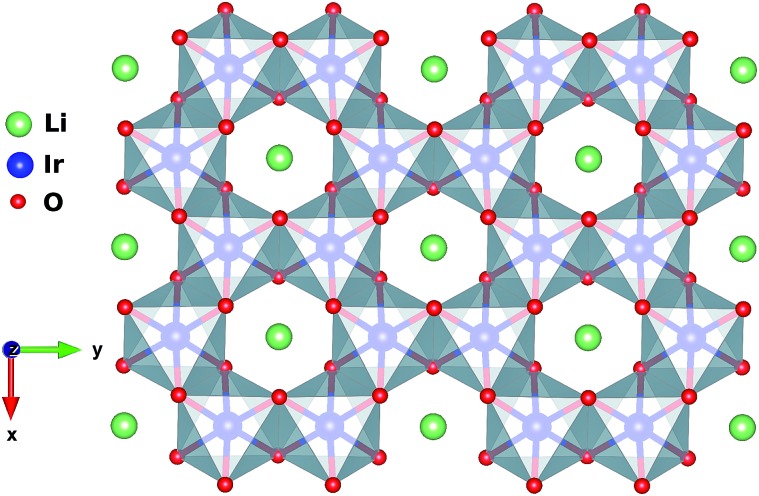
Honeycomb-like layer in H_3_LiIr_2_O_6_. A Li ion is present at the center of each hexagonal ring of edge-sharing IrO_6_ octahedra.

The prospect of realizing spin-liquid (SL) ground states in layered honeycomb materials with strong spin–orbit interactions^1^,^2^ has triggered intense research activity in relation to these lattice systems. Quantum SLs are of particular interest in connection with properties such as protection of quantum information and the emergence of Majorana fermions. On a honeycomb lattice (Fig. 1), the essential ingredient for the formation of a quantum SL state is the so-called Kitaev coupling (*K*) between nearest-neighbor (NN) magnetic sites, a bond-dependent Ising-like exchange^1^,^2^ that must be large enough as compared to the more conventional NN Heisenberg *J*. It reaches quite robust values for d^5^ electron configurations in iridium honeycomb oxides such as Na_2_IrO_3_ (ref. 3 and 4) but also in the ruthenium halide RuCl_3_.^5^,^6^ In the latter, a SL phase is realized by applying an external magnetic field.^7^,^8^

**Fig. 2:**
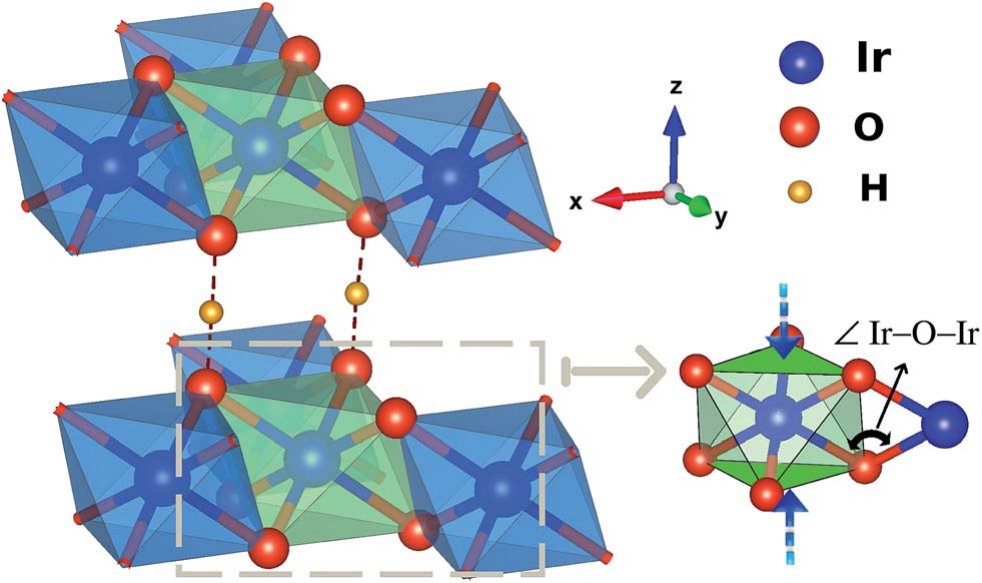
Ir–O bonds in H_3_LiIr_2_O_6_. Ir ions belonging to two adjacent honeycomb planes are displayed, along with two inter-layer H sites. The IrO_6_ octahedra are trigonally compressed: triangular facets above and below the honeycomb planes are closer to each other. This makes the Ir–O–Ir angles larger than 90°.

**Fig. 3:**
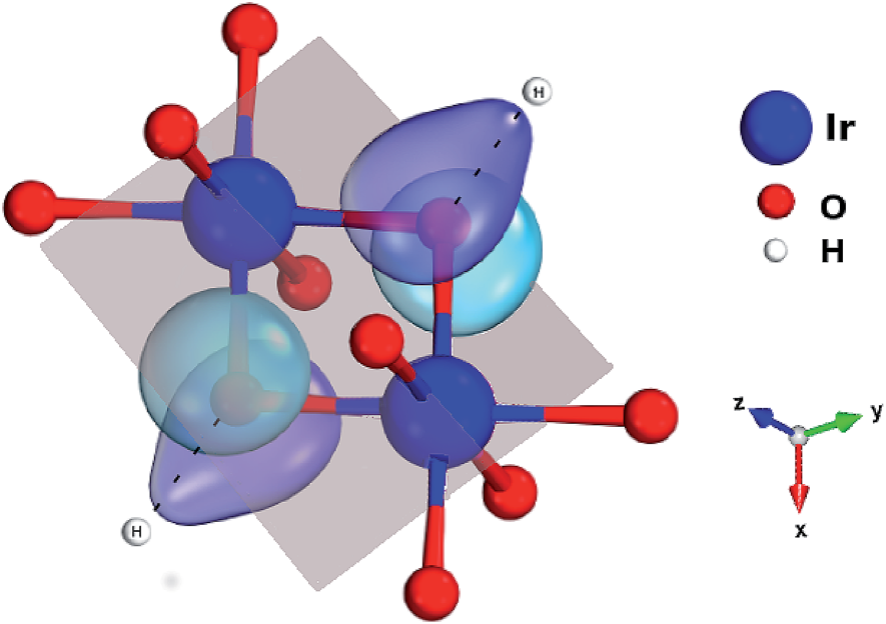
Ir_2_O_2_ plaquette and the O 2p orbitals mediating superexchange on that plaquette. There are two 5d t_2g_ components per Ir site (not shown) having a direct, π-type overlap with the O 2p orbitals depicted in the figure. Adjacent H's strongly affect the d–p overlap matrix elements, through unfavorable polarization of the bridging ligand 2p functions.

One peculiar prediction on the computational side is an enhancement of the Kitaev coupling *K* at large Ir–O–Ir bond angles.^9^ The Ir–O–Ir bond angles are 90° for cubic edge-sharing octahedra, but in most honeycomb compounds they reach larger values due to trigonal compression of the oxygen cages (see Fig. 2). The largest Ir–O–Ir bond angles so far have been reported for H_3_LiIr_2_O_6_, nearly 100°.^10^ Interestingly, Kitagawa *et al.* inferred a SL ground state for this material.^11^ We examined in this context the Kitaev interactions of H_3_LiIr_2_O_6_ but for ideal stacking of the honeycomb layers found rather modest *K* values as compared to, *e.g.*, Na_2_IrO_3_ (ref. 3 and 4) and earlier predictions for 100° Ir–O–Ir angles.^9^ In an attempt to reconcile these apparently contradicting sets of computational results for large Ir–O–Ir bond angles, we addressed in detail the effect of having a single adjacent H site for each O ion and only ‘vertical’ O–H–O paths for the simplest stacking pattern.^10^ We establish that the axial potential created through this kind of O–H coordination polarizes and bends towards the O–H link the O 2p orbitals bridging between Ir t_2g_ components orthogonal to the Ir_2_O_2_ plaquette (see Fig. 3). Such polarization effects are absent in Li_2_IrO_3_ and Na_2_IrO_3_, for coordination with several inter-layer cations of the O sites, but for ideal stacking in H_3_LiIr_2_O_6_ (ref. 10) they disrupt Ir–O–Ir electron hopping and consequently reduce the Kitaev exchange.

Numerical tests in which the two H ions next to an Ir_2_O_2_ plaquette are simply removed yield an impressively large ferromagnetic (FM) |*K*| value of 40 meV. Given the experimental indications for a SL ground state in H_3_LiIr_2_O_6_,^11^ these computational findings provide additional support for the existence of stacking faults^10^ and H-ion disorder in this system, since larger *K*'s should in principle make the quantum SL more likely. Moreover, our results provide valuable guidelines for the rational design of Kitaev quantum magnets, indicating that electrostatic effects involving inter-layer species are very important and that the largest *K* values come with a more isotropic distribution of inter-layer cations around a given ligand.

## Quantum chemistry exchange couplings

Each IrO_6_ octahedron shares edges with three NN IrO_6_ octahedra such that the Ir sites frame a two-dimensional (2D) honeycomb lattice in H_3_LiIr_2_O_6_. Similar to its parent compound α-Li_2_IrO_3_, a Li ion is present at the center of each Ir_6_ hexagon; an essential structural difference is the replacement of Li species between adjacent honeycomb layers by H ions. The octahedral ligand coordination of the Ir sites gives rise to a large gap between the t_2g_ and e_g_ 5d levels. The leading ground-state configuration is therefore ^2^T_2g_ (t52g), with all five valence electrons in the t_2g_ orbitals. This corresponds to an *L* = 1 orbital angular momentum^12^ and *via* strong spin–orbit coupling yields a magnetically active *j*_eff_ = 1/2 ground-state doublet and lower-lying, occupied *j*_eff_ = 3/2 states.^2^,^12^ The remarkable feature displayed by NN t52g ions with strong spin–orbit interactions and edge-sharing connectivity of the encircling ligand cages is a large Ising-like interaction *KS[combining tilde]*γ*iS[combining tilde]*γ*j* between spin components perpendicular to a given M_2_L_2_ plaquette (L stands for a ligand, see Fig. 2 and 3 for more details). On a honeycomb network of ML_6_ octahedra this Ising-like coupling is bond dependent, *i.e.*, the index *γ*(*γ* ∈ {*x*,*y*,*z*}) is different for each of the three M–M links emerging out of a given metal site M. Following Kitaev's conceptualization and initial analysis,^1^ strong interactions of this type were suggested to be realized in Ir oxide compounds such as Na_2_IrO_3_ and Li_2_IrO_3_.^2^ To derive the strength of *K* in the related material H_3_LiIr_2_O_6_, and also of additional symmetric anisotropic terms and of the isotropic Heisenberg component, we rely on *ab initio* many-body computational schemes from quantum chemistry. The NN superexchange is analyzed on clusters of two edge-sharing IrO_6_ octahedra, embedded in an effective field that models the remaining part of the crystalline lattice.

Pairs of adjacent IrO_6_ octahedra of two slightly different types were reported on the basis of X-ray diffraction data,^10^ with Ir–O–Ir bond angles of either 99.0 or 99.8 degrees. Since the difference between these two values is rather small, we consider in our calculations a slightly idealized crystal structure with ‘averaged’ Ir–O–Ir bond angles of 99.4° and Ir–Ir bond lengths of 3.08 Å. For two NN octahedra, the *C*_2h_ point-group symmetry allows two extra, symmetric off-diagonal exchange terms in addition to the isotropic Heisenberg and anisotropic Kitaev components.^4^ The effective spin Hamiltonian for a pair of pseudospins at NN Ir sites *i* and *j* can then be written as1
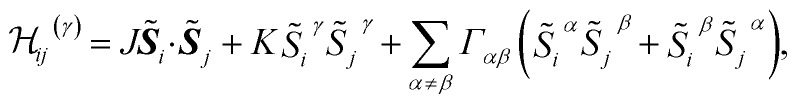
where *α*, *β* ∈ {*x*, *y*, *z*}. We use in the following a local Kitaev reference frame^2^,^4^ in which the *z* axis is perpendicular to the Ir_2_O_2_ plaquette (*i.e.*, *γ* = *z*); given the *C*_2h_ symmetry, *Γ*_*zx*_ = –*Γ*_*yz*_ in this setting. The NN magnetic couplings discussed in the following are derived by mapping^5^,^13^ the *ab intio* quantum chemistry data onto such an effective spin Hamiltonian.

Multiconfigurational wavefunctions were obtained to this end by complete-active-space self-consistent-field (CASSCF) calculations,^14^ using an active space consisting of six t_2g_ orbitals at the two NN Ir sites (see the Methods section for additional details). Post-CASSCF, we also performed multireference configuration-interaction (MRCI) computations^14^ accounting for single and double excitations out of the Ir 5d t_2g_ and bridging-ligand 2p orbitals. The reference CASSCF wavefunctions were variationally optimized for the lowest nine singlets and nine triplets, which subsequently entered the spin–orbit treatment to yield 36 spin–orbit states. The lowest four of these states define the actual magnetic problem of two interacting pseudospin-1/2 sites, as pointed out by Jackeli and Khaliullin,^2^ and were mapped onto the effective spin Hamiltonian (1), in order to derive the NN effective exchange couplings. The other 32 levels lie at significantly higher energy, ≧0.5 eV, and are associated with *j* ≈ 3/2 to *j* ≈ 1/2 excitations,^2^,^12^ as shown by joint resonant inelastic X-ray scattering (RIXS) and quantum chemistry investigations.^15^,^16^ This large gap between the two sets of spin–orbit states, Ir *j* ≈ 1/2 and *j* ≈ 3/2, ensures that decoupling these two sectors in the mapping procedure is a safe approximation.^2^ Such a strategy is also widely applied in the context of f electron superexchange.^17^ All calculations were performed using the MOLPRO quantum chemistry package.^18^ Using the same methodology, MRCI exchange couplings in good agreement with experimental data were reported earlier for square-lattice iridates,^13^,^19^ the pyrochlore Sm_2_Ir_2_O_7_,^20^ and the perovskite CaIrO_3_.^21^,^22^

## Results and discussion

The M–L–M angle is one of the key factors in tuning the magnitude of the Kitaev and Heisenberg components: as pointed out in ref. 9 and 23, larger angles lead to larger anisotropic interactions in honeycomb t52g oxides. Since in comparison to the related iridates Na_2_IrO_3_ and α-Li_2_IrO_3_, the Ir–O–Ir angles are on the larger side in H_3_LiIr_2_O_6_ (99–100°),^10^,^11^ one would expect Kitaev couplings of larger magnitude in this system. For the other iridates, FM values in the range of 15–20 meV were found by MRCI;^4^,^9^ however, for angles close to 100° and ideal stacking of successive honeycomb layers, we here compute a FM *K* value of only 10.9 meV (see Table 1). This suggests some subtle differences between interactions in H_3_LiIr_2_O_6_ and in, *e.g.*, Na_2_IrO_3_, the identification of which constitutes a main purpose of this paper. The actual splittings among the lowest four ‘magnetic’ levels along with the other effective coupling constants are also listed in Table 1, as obtained by both CASSCF and MRCI calculations.

**Table 1:** Splittings among the lowest four spin–orbit states, mapped onto the eigenstates of the effective model defined by (1), and the resulting effective exchange couplings for two edge-sharing IrO_6_ octahedra (all values in meV); a slightly idealized crystal structure with averaged bond lengths and bond angles was used (see the text)

Magnetic splittings	CASSCF + SOC	MRCI + SOC
*Ψ*_2_ = (↑↑ + ↓↓)/√2	0.0	0.0
*Ψ*_3_ = (↑↑ – ↓↓)/√2	0.3	1.1
*Ψ*_S_ = (↑↓ – ↓↑)/√2	3.3	4.0
*Ψ*_1_ = (↑↓ + ↓↑)/√2	4.9	7.1

Effective couplings	*K*	*J*	*Γ*_*xy*_	*Γ*_*yz*_ = –*Γ*_*zx*_
CASSCF + SOC	–6.8	–2.5	–0.4	–1.5
MRCI + SOC	–10.9	1.8	–0.6	–2.0

We note that results similar to those provided in Table 1 are obtained by spin–orbit MRCI^24^ when considering the presence of two, structurally different Ir–Ir links (referred to as B1 and B2) of the type proposed in ref. 10 and 11, with somewhat different bond angles and bond lengths between the B1 and B2 blocks of NN IrO_6_ octahedra. Estimates for *J*, *K* and the off-diagonal couplings were also derived in very recent studies on the basis of density-functional computations.^25^,^26^ *K*, for example, becomes significantly stronger in the latter investigations, by a few meV in ref. 25 and by a factor of nearly 2 in ref. 26.

### Interlayer electrostatics, impact on Kitaev exchange

From a structural point of view, two groups of compounds can be identified within the family of 5d^5^ honeycomb iridates: Na_2_IrO_3_ and α-Li_2_IrO_3_, where each cation in-between the honeycomb-like sheets has six adjacent oxygen sites, and Cu_2_IrO_3_ (ref. 27) and H_3_LiIr_2_O_6_,^10^,^11^ displaying O–M′–O contacts with just two oxygen NNs for each inter-layer cation M′ if stacking faults are absent.^10^ For the latter type of interlayer connectivity, coordination by a single M′ cation of each O ligand implies an out-of-plane field and polarization of the O 2p valence electronic cloud along the O–M′–O axis. We quantified the effect of such anisotropic out-of-plane fields in an additional set of calculations, where the two hydrogen ions next to the two O sites shared by Ir NNs (see Fig. 2) were simply taken away.

We find that removal of two H's next to the bridging ligands results in a nearly four-fold increase of the Kitaev exchange between in-plane NN *S[combining tilde]* = 1/2 sites: from ≈ –11 meV (see Table 1), it now reaches –40 meV in the spin–orbit MRCI calculations. The proximity of the positive H ions is associated with two different effects on the in-plane spin–spin interactions: (i) the ‘bare’ effect of the H-ion Coulomb potential on on-site orbital energies and intersite hopping matrix elements and (ii) O 2p orbital polarization effects that can additionally affect the Ir–O–Ir orbital overlaps and therefore, once again, the intersite hoppings. To determine which is the dominant mechanism, we performed extra computations at the CASSCF level. NN CASSCF magnetic couplings obtained for a cluster where each of the H ions next to a bridging ligand is removed and the associated ionic charge is redistributed within the embedding are listed on the first line in Table 2. This *K* value, –27.4 meV, corresponds to the CASSCF states used as a reference in the configuration interaction calculations leading to the MRCI result *K* = –40 meV. In a second step, we modeled those two H ions as simple point charges (PCs) but did not allow relaxation of the cluster orbitals. In other words, multiconfigurational computations were performed without orbital reoptimization, which are also referred to as frozen-orbital, CASFO calculations. The exchange interactions are somewhat suppressed due to the presence of the nearby positive charge (see the second line in Table 2). However, allowing the orbitals to fully relax, *i.e.*, to react to the axial potential generated by the adjacent unit PCs, results in a much more drastic reduction of the NN magnetic couplings (see the third row in Table 2). This step by step analysis makes it clear that orbital polarization in response to the electrostatic potential induced by the inter-layer H cations is the primary cause of the lower NN interaction constants listed in Table 1.

**Table 2:** Effect of inter-layer species on NN magnetic couplings (in meV). The two H ions next to the bridging O ligands are first removed (first line) and subsequently placed as point charges (lowest two lines)

	*K*	*J*	*Γ*_*xy*_	*Γ*_*yz*_ = –*Γ*_*zx*_
CASSCF, no H NNs	–27.4^*a*^	–7.1	2.1	–4.0
CASFO, PC H NNs	–21.8	2.1	3.0	–2.9
CASSCF, PC H NNs	–6.9	0.8	0.5	–1.3

^*a*^The corresponding MRCI value is *K* = –40 meV (see the text).

A large amount of stacking faults was evidenced in H_3_LiIr_2_O_6_,^10^ most probably related to the rare situation in which H is bridging two adjacent O sheets. Having the hydroxyl bond in mind, it has been pointed out that an alternative way of writing the chemical formula of this compound is LiIr_2_O_3_(OH)_3_.^10^ An idealized picture arising from this formula is then that of alternating [LiIr_2_O_6_]^3–^ and [LiIr_2_(OH)_6_]^3+^ honeycomb-like layers (or slabs), the latter with all bridging O's replaced by hydroxyl groups, as in the related material Li_2_Pt(OH)_6_.^28^ The weak bonding between layers and the inherent stacking disorder is even better highlighted in such a representation, the frail hydrogen bonds O–H···O being more apparent. In this context,^10^,^28^ our results strongly suggest the existence of both ‘ideally stacked’^11^ (*i.e.*, weak, see Table 1) and ‘fault-present’^10^ (*i.e.*, strong, see Table 2) exchange couplings in this system, which then makes the modelling of the extended magnetic lattice more complicated.

While the role of inter-layer ionic species is analyzed here for H_3_LiIr_2_O_6_, ongoing work^29^ yields similar results for the copper iridate Cu_2_IrO_3_,^27^ displaying similar O–M′–O interlayer contacts. These data nicely complement earlier findings concerning the sensitivity of various effective magnetic couplings to the position and charge of secondary/tertiary cations in oxide compounds.^13^,^30^ Quantities addressed in the earlier investigations were on-site parameters such as *g* factors^13^ and zero-field splittings.^30^ Here it is explicitly shown that also the intersite magnetic couplings can be adjusted by using electrostatic effects involving ionic species beyond the crystalline region (*i.e.*, ‘beyond’ the bridging ligands) that is commonly assumed to be of relevance.

### Angle dependence, the Kitaev limit

**Fig. 4:**
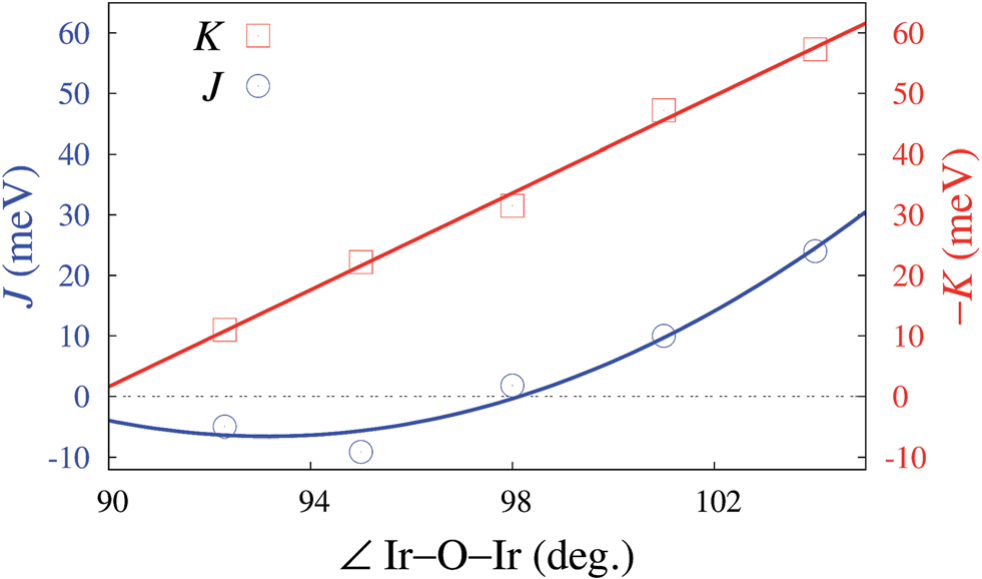
NN Kitaev and Heisenberg couplings for variable Ir–O–Ir angles in model *C*2/*m*-type structures, spin–orbit MRCI results. The NN Ir–Ir distance is set to 3.08 Å and the Ir–O bond lengths are for a given Ir–O–Ir angle all the same. The variation of the Ir–O–Ir angle is the result of gradual trigonal compression. Curves are drawn just as guides for the eye.

Looking at tuning structural parameters having to do with the honeycomb-like [LiIr_2_O_6_]^3–^ slab, we further determined the evolution of both *K* and *J* with gradually modifying the Ir–O–Ir bond angles. We focused on atomic configurations where the two H cations next to the bridging ligands are removed since such arrangements are found to yield very large *K*'s and are additionally likely to occur in the actual material. To maintain overall neutrality, the formal ionic charge associated with the two H's was again redistributed within the embedding; the Ir–Ir distance was fixed at 3.08 Å while a variable Ir–O–Ir bond angle was achieved through gradual trigonal distortion of the IrO_6_ octahedra (see Fig. 2). The resulting *K*'s and *J*'s are shown in Fig. 4. The remarkable feature is that for bond angles close to 98°, *J* → 0. That is, a purely anisotropic effective magnetic model can be realized according to our calculations for ≈98°, with FM Kitaev coupling constants as large as 31 meV. This critical angle defining on the computational side the Kitaev ‘limit’ is actually not so far from the bond angles reported for H_3_LiIr_2_O_6_ (ref. 11)—according to the quantum chemistry results, only small structural modifications would be required for reaching the regime of vanishing *J*. Interestingly, it turns out that not only the M–L–M angle constitutes here a tuning knob but also the interatomic distances; especially in the vicinity of the critical value *θ*_c_, *J* can be reduced towards zero by also varying the bond lengths, *via* tensile strain for instance.^31^

### Exchange between honeycomb planes

A recent theoretical model^32^ attempting to explain the experimentally observed magnetic properties of H_3_LiIr_2_O_6_ (ref. 11) assumes interlayer isotropic couplings as large as 10 meV. To verify this assumption we carried out an additional set of calculations, on a cluster having as the active region two IrO_6_ octahedra that belong to adjacent honeycomb layers and are connected through double O–H–O paths as proposed in ref. 11 (see Fig. 2). A rather similar kind of connectivity is in fact also encountered in the 5d^5^ triangular-lattice system Ba_3_IrTi_2_O_9_;^33^ it is often referred to as double-edge connectivity in the case of Ba_3_IrTi_2_O_9_, since there is no cation in-between O sites of NN octahedra in that material.

Estimates for double O–O bridges in Ba_3_IrTi_2_O_9_ yield interaction strengths in the range of a few meV, for both Heisenberg and Kitaev exchange.^33^ For O–H–O links in H_3_LiIr_2_O_6_, we compute by spin–orbit MRCI effective Heisenberg and Kitaev couplings in the region of 1 meV. This suggests that an interaction strength of ≈10 meV as assumed in ref. 32 for the interlayer Heisenberg exchange is rather excessive.

## Conclusions

In summary, linear interlayer linkage with oxygen and inter-layer cation sites aligned in three-center bonds perpendicular to the magnetic planes reduces orbital overlap along Ir–O–Ir paths within the honeycomb-like LiIr_2_O_6_ layers and the Kitaev couplings, through polarization and bending towards the vertical O–H–O axis of the Kitaev-active O 2p orbital. Here we demonstrate this for stacked [LiIr_2_O_6_]^3–^ honeycomb sheets with O–H–O linear linkage but similar effects should govern the magnetism of related compounds such as Cu_2_IrO_3_.^27^ For the latter, the interlayer O–Cu–O linear bonds are also referred to as dumbbell bonds. Interestingly, for the lighter inter-layer cation, a large amount of stacking faults has been experimentally determined.^10^ Our computational findings indicate that randomness in stacking of the honeycomb layers and H-ion vacancies would remove the axial cationic potential at least for part of the O ligands, which yields an unparalleled Kitaev interaction strength of –40 meV for Ir–O–Ir angles of ≈100°, larger by factors of 2–3 as compared to the honeycomb Kitaev–Heisenberg material Na_2_IrO_3_ (ref. 4) and 6 in comparison to RuCl_3_.^5^ Our results therefore provide valuable insights into the magnetism of the SL candidate H_3_LiIr_2_O_6_ (ref. 11) and additionally simple rules for achieving the Kitaev SL ground state in other honeycomb iridates: large Ir–O–Ir bond angles in the region of 98°, since *J* → 0 in that range, and coordination of the honeycomb-plane ligands by more than one inter-layer cation. Both features, the nature and the position of ionic species next to the honeycomb sheets and the size of the Ir–O–Ir bond angles, can be in principle more effectively tailored in stacked heterostructures. First steps are being made in this direction^34^,^35^ and it becomes apparent that this research area holds much potential for engineering magnetic couplings in Kitaev–Heisenberg systems.

## Methods

The magnetic exchange couplings between NN Ir sites were derived by calculations on embedded clusters having two edge-sharing octahedra (Ir_2_O_10_ units) as the central region. To properly describe multiorbital physics within the Ir t_2g_ sector, we rely on a CASSCF scheme^14^ as the starting point. In this frame, the most obvious choice for the active multiconfigurational space is that based on having six t_2g_ orbitals (three t_2g_ orbitals per Ir site) and ten electrons (two holes in the Ir t_2g_ channel). Since all possible electron configurations are here accounted for, we naturally describe in this way superexchange processes involving virtual excited states of t42g–t62g type. Additional intersite excitations, both M–M (t_2g_–e_g_) and L–M, enter our correlation treatment in the subsequent MRCI calculations.^36^,^37^ Spin–orbit couplings were computed according to the methodology described in ref. 38. To model the finite charge distribution in the immediate neighborhood, the adjacent four octahedra were also explicitly included in the calculations. Energy-consistent relativistic pseudopotentials along with quadruple-zeta basis functions^39^ were used for the Ir ions of the central unit. All-electron basis sets of quintuple-zeta quality^40^ were employed for the bridging O ligands while all-electron basis sets of triple-zeta quality^40^ were used for the other O anions within the two-octahedron central region. Ir^4+^ sites belonging to octahedra adjacent to the reference unit were described as closed-shell Pt^4+^ t62g species, using relativistic pseudopotentials and valence triple-zeta basis functions.^39^ Ligands of these adjacent octahedra that are not shared with the central reference unit were modeled with minimal all-electron atomic-natural-orbital basis sets.^41^ For the Li NNs we employed total-ion effective potentials with a single s valence basis function^42^ while two s and one p valence basis functions were used for the H NNs.^43^

To extract the effective magnetic couplings, the lowest four spin–orbit states associated with the t52g–t52g manifold in the quantum chemical treatment were mapped onto the eigenstates of the effective spin Hamiltonian (1). To evaluate all symmetry-allowed coupling constants in (1), we additionally considered the Zeeman coupling term 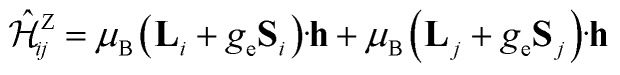 For the latter, all required matrix elements are available in the MOLPRO output data, *i.e.*, the expectation values of the orbital angular momentum (**L**_*i*_, **L**_*j*_) and spin (**S**_*i*_, **S**_*j*_) operators (see also ref. 5 and 13 for additional details, in particular, the matrix elements listed in Tables 5 and 6 in ref. 5). The one to one correspondence between Hamiltonian matrix elements obtained at the *ab initio* and effective-model levels allows us to evaluate all coupling constants involved in (1).^5^,^13^,^20^

Additional quantum chemistry computations were performed to investigate the strength of magnetic exchange between Ir sites belonging to adjacent honeycomb layers, connected *via* double O–H–O pathways. For this set of calculations, an Ir_2_O_12_H_2_ unit was used as the central region. The six NN IrO_6_ octahedra directly coordinating the two reference octahedra (three NN octahedra around each ‘central’ octahedron) plus eight H NNs lying close to the central fragment were also included in the calculations, for better representation of the immediate neighborhood. The two Ir ions in the central unit were represented by energy-consistent relativistic pseudopotentials along with triple-zeta basis functions.^39^ All-electron basis sets of quintuple-zeta quality^40^ were employed for the four O ligands on the O–H–O contacts while all-electron basis sets of triple-zeta quality^40^ were used for the remaining O ions within the central region; triple-zeta basis sets^40^ were applied for the two bridging H's. Ir^4+^ sites belonging to octahedra adjacent to the central region were described as closed-shell Pt^4+^ t62g species, using relativistic pseudopotentials and valence double-zeta basis functions.^39^ Ligands of these adjacent octahedra that are not shared with the central reference unit were modeled with minimal all-electron atomic-natural-orbital basis sets.^41^ For the Li NNs we employed total-ion effective potentials with a single *s* valence basis function^42^ while double-zeta basis functions were utilized for the remaining H ions.^40^ PC embeddings were used in all calculations, as in earlier quantum chemistry investigations of honeycomb iridates.^4^,^9^,^15^

## Conflicts of interest

There are no conflicts to declare.
